# Embryonic cranial cartilage defects in the *Fgfr3* ^*Y367C*^ ^*/+*^ mouse model of achondroplasia

**DOI:** 10.1002/ar.25327

**Published:** 2023-09-25

**Authors:** Susan M. Motch Perrine, Nishchal Sapkota, Kazuhiko Kawasaki, Yejia Zhang, Danny Z. Chen, Mizuho Kawasaki, Emily L. Durham, Yann Heuzé, Laurence Legeai‐Mallet, Joan T. Richtsmeier

**Affiliations:** ^1^ Department of Anthropology The Pennsylvania State University University Park Pennsylvania USA; ^2^ Department of Computer Science and Engineering University of Notre Dame Notre Dame Indiana USA; ^3^ Department of Pediatrics, Division of Human Genetics Children's Hospital of Philadelphia Philadelphia Pennsylvania USA; ^4^ Univ. Bordeaux, CNRS, Ministère de la Culture PACEA, UMR 5199 Pessac France; ^5^ Université de Paris Cité Imagine Institute, Laboratory of Molecular and Physiopathological Bases of Osteochondrodysplasia, INSERM UMR 1163 Paris France

**Keywords:** achondroplasia, chondrocranium, craniofacial, FGFR3, Meckel's cartilage, morphology, skeletal dysplasia

## Abstract

Achondroplasia, the most common chondrodysplasia in humans, is caused by one of two gain of function mutations localized in the transmembrane domain of fibroblast growth factor receptor 3 (FGFR3) leading to constitutive activation of FGFR3 and subsequent growth plate cartilage and bone defects. Phenotypic features of achondroplasia include macrocephaly with frontal bossing, midface hypoplasia, disproportionate shortening of the extremities, brachydactyly with trident configuration of the hand, and bowed legs. The condition is defined primarily on postnatal effects on bone and cartilage, and embryonic development of tissues in affected individuals is not well studied. Using the *Fgfr3* ^*Y367C/+*^ mouse model of achondroplasia, we investigated the developing chondrocranium and Meckel's cartilage (MC) at embryonic days (E)14.5 and E16.5. Sparse hand annotations of chondrocranial and MC cartilages visualized in phosphotungstic acid enhanced three‐dimensional (3D) micro‐computed tomography (microCT) images were used to train our automatic deep learning‐based 3D segmentation model and produce 3D isosurfaces of the chondrocranium and MC. Using 3D coordinates of landmarks measured on the 3D isosurfaces, we quantified differences in the chondrocranium and MC of *Fgfr3* ^*Y367C/+*^ mice relative to those of their unaffected littermates. Statistically significant differences in morphology and growth of the chondrocranium and MC were found, indicating direct effects of this *Fgfr3* mutation on embryonic cranial and pharyngeal cartilages, which in turn can secondarily affect cranial dermal bone development. Our results support the suggestion that early therapeutic intervention during cartilage formation may lessen the effects of this condition.

## 1 INTRODUCTION

Achondroplasia (ACH) is the most frequent skeletal dysplasia associated with disproportionate short stature (Horton et al., 2007). ACH occurs in ~1 in 10,000 to 30,000 live births, regardless of sex or genetic ancestry (Savarirayan et al., 2022). The molecular basis of ACH was elucidated in 1994 as a heterozygous pathogenic variant in the fibroblast growth factor receptor 3 (*FGFR3*) gene on chromosome 4p16.3 (Rousseau et al., 1994; Shiang et al., 1994). The resulting FGFR3 gain of function mutation in which two different substitutions at nucleotide 1138 both result in the amino acid change p.Gly380Arg (Horton et al., 2007) inhibits the proliferation and differentiation of chondrocytes in the growth plate (Legeai‐Mallet et al., 2004; L'Hôte & Knowles, 2005). Osseous abnormalities associated with ACH have been described in all endochondral bones, including those of the skull (ethmoid, parts of the sphenoid, petrous temporal, and most of the occipital). Additionally, the endochondral cranial base (usually thought of as consisting of the basi‐occipital and sphenoid but can also include the presphenoid, ethmoid, and petrous temporals) of affected individuals has been described as short and narrow, encasing less than the usual portion of the brain and resulting in an abnormally large cranial vault with midfacial hypoplasia and frontal bossing (Crawford et al., 1978; Rocco et al., 2014). The mandible, which is formed principally of intramembranous bone, is disproportionately large (Crawford et al., 1978). The large brain, specifically due to increased cerebellar volume (Pascoe et al., 2020) is associated with a neurocranium that is large relative to a small body and may cause developmental delay in raising the head, sitting, and standing. Additional oro‐facial findings in ACH patients include posterior crossbite, anterior open bite, prognathic mandible, and retrognathic maxilla with reverse overjet and high‐arched palate. Delayed eruption of teeth and oligodontia due to altered bone growth that may contribute to affected masticatory and respiratory functions are also common in ACH patients (Sforza et al., 2023). ACH is characterized by a multitude of medical, functional, and psychosocial challenges throughout life. Awareness and appropriate management of these challenges are key to facilitating optimal outcomes and quality of life of ACH individuals and their families (Savarirayan et al., 2022).

Mouse models of ACH have provided valuable insight into the impact of constitutive activation of the FGFR3 protein on development of multiple tissues. *Fgfr3* ^*Y367C/+*^ mice carrying a heterozygous Y367C amino acid substitution in FGFR3 corresponding to the human Y373C mutation were previously shown to exhibit a penetrant inner ear defect with significantly elevated auditory brainstem response threshold in addition to severe dwarfism (Pannier et al., 2009). At birth, the *Fgfr3* ^*Y367C/+*^ mice exhibited skeletal dysplasia that worsened over time. Bowed tibias, fibulas, ulnas, and radii in addition to reduced femoral length and reduced caudal vertebrae length, a narrow trunk, short ribs, and macrocephaly were seen in male and female mutant mice (Pannier et al., 2009). Histological analyses of epiphyseal growth plates showed disorganization, with shortened chondrocyte columns and reduced number and size of hypertrophic chondrocytes (Pannier et al., 2009). Radiographs and high‐resolution computed tomography of postnatal mice revealed a dome‐shaped skull, shortened cranial base, hypoplastic midface, prognathic mandible, abnormal orientation of the semicircular canals and cochlea, anteriorly displaced foramen magnum, and poorly developed temporal bones as well as delayed ossification of inner ear bones (Pannier et al., 2009).

Dysmorphology of the lower jaw in ACH has been analyzed in humans and mice, while inhibition of FGFR3 signaling has been studied in the chick. Studies of chick mandibles suggested FGFR3 signaling is required for the elongation of Meckel's cartilage (MC), while FGFR2 and FGFR3 are required for intramembranous ossification of mandibular bones (Havens et al., 2008). Blocking FGFR3 signaling in the chick mandible affected the proliferation, survival, and differentiation of chondroblasts, providing evidence for an essential role of FGF/FGFR3 signaling during morphogenesis and elongation of MC (Havens et al., 2008). Stage and region‐dependent inhibition of FGFR3 signaling were also demonstrated in chick MC and mandibular bones (Mina & Havens, 2007). Decreased mandibular length has been reported in ACH children and adult patients (Morice et al., 2018). Mandibular length was also decreased in *Fgfr3* ^*Y367C/+*^ mice relative to *Fgfr3* ^*+/+*^ littermates at embryonic day (E)16.5, E18.5, postnatal day (P)0, and P21 (Duplan et al., 2016). Chondrocyte homeostasis was found to be disturbed in MC of E16.5 *Fgfr3* ^*Y367C/+*^ mice, with disrupted chondrocyte differentiation. The size of the hypertrophic chondrocyte zone relative to the total size of MC was reduced, as was the size of individual hypertrophic chondrocytes in *Fgfr3* ^*Y367C/+*^ mice at E16.5, while more cells were proliferating in MC in mice carrying the mutation (Duplan et al., 2016). There was an increase in the number of FGFR3‐positive cells in MC at E16.5, similar to what was reported in the growth plate of the same mouse line (Jonquoy et al., 2012; Komla‐Ebri et al., 2016), and a 29% increase in the size of MC as measured in sagittal histological sections at E16.5 (Duplan et al., 2016).

Although we understand the disruption of the growth plate in achondroplasia, the condition is primarily defined on the obvious effects on postnatal bones and cartilage. Embryonic development of tissues in affected individuals is less well studied. Using the *Fgfr3* ^*Y367C/+*^ mouse model of ACH, we investigated the developing chondrocranium and MC (part of the pharyngeal skeleton) at embryonic day (E)14.5 and E16.5.

## 2 MATERIALS AND METHODS

### 2.1 Sample

*Fgfr3* ^*Y367C/+*^ mice were generated by a previously described protocol (Pannier et al., 2009). Affected mice ubiquitously express an *Fgfr3* variant encoding the cytosine residue at the 367th amino acid (Y367C). Mice were raised and maintained on a C57BL/6J background. Litters of mice consisting of those carrying the variant of interest (*Fgfr3* ^*Y367C/+*^) and unaffected (*Fgfr3* ^*+/+*^) littermates were collected into cold phosphate‐buffered saline (PBS) on ice at E14.5 and E16.5 at Imagine institute (Paris, France) following appropriate animal care and use protocols. Specimens were genotyped and shipped to The Pennsylvania State University for staining and imaging. The sample consisted of two litters at each age produced via timed matings (9 E14.5 specimens [4 *Fgfr3* ^*+/+*^; 5 *Fgfr3* ^*Y367C/+*^] and 11 E16.5 specimens [6 *Fgfr3* ^*+/+*^; 5 *Fgfr3* ^*Y367C/+*^]) and specimens were staged using the eMOSS system (Musy et al., 2018) to ensure they were within ±6 h of expected age.

### 2.2 Microcomputed tomography (MicroCT) data acquisition

All scans were acquired at the Penn State Center for Quantitative Imaging using the General Electric v|tome| × L300 nano/microCT system. E14.5 and E16.5 specimens were stained with phosphotungstic acid (PTA) following previously described protocols (Lesciotto et al., 2020). PTA enhanced microCT scans were acquired on E14.5 specimens using the 180 kV tube at 100 kV and 65 μA with one 0.2 mm aluminum filter on the detector and a voxel size of 6.49 μm. PTA‐enhanced microCT scans of E16.5 specimens were acquired using the 180 kV tube at 100 kV and 70 μA, with one 0.2 mm aluminum filter on the detector and a voxel size of 8.5 μm. Raw data were reconstructed using Phoenix datos| × 2 reconstruction software as 32‐bit .vol files.

### 2.3 Stack post‐processing

32‐bit .vol data were cropped, reoriented into anatomical position, and transformed to 16‐bit image stacks using Dragonfly (Object Research Systems, Montréal, Québec, Canada). Histogram leveling and LUT adjustment were performed in Fiji (Schindelin et al., 2012). All specimens were treated similarly.

### 2.4 Cartilage segmentation

**FIGURE 1:**
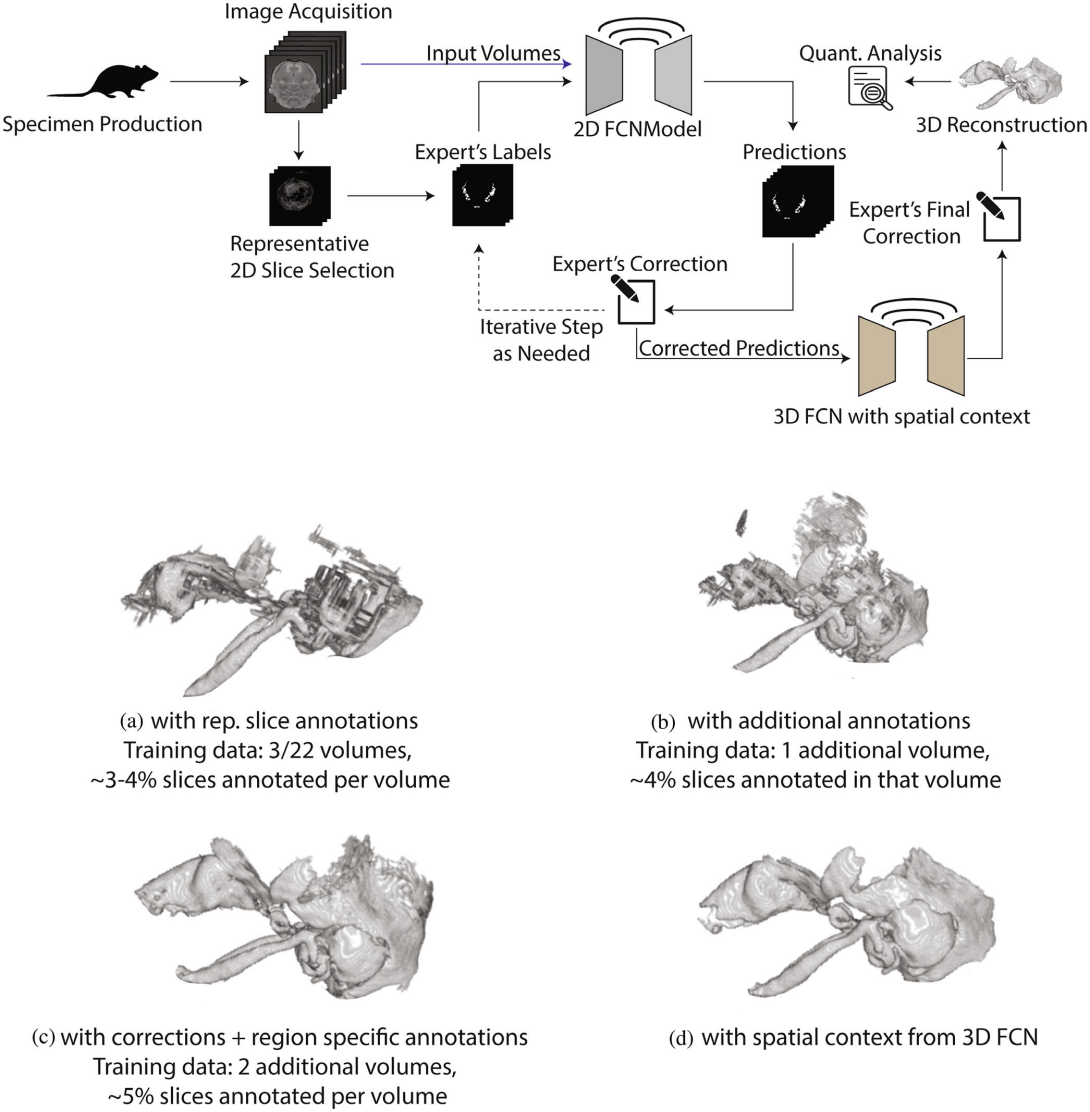
Coordinated cartilage segmentation approach. Data processing pipeline (top): Specimens were acquired at the Université de Paris Cité Imagine Institute (Paris, France). PTA‐enhanced microCT acquisition and reconstruction were performed at the Penn State Center for Quantitative Imaging (University Park, PA, USA). Machine learning segmentation was performed at the University of Notre Dame (Notre Dame, IN, USA). First, representative 2D slices were selected for expert annotations. Expert annotations were performed at the Pennsylvania State University (University Park, PA, USA). A 2D FCN model was trained using the annotated slices (the training set consists of only those volumes for which annotated were provided) to generate segmentation masks. Through iterative refinement, experts corrected the masks and provided additional annotations on error‐prone regions (if needed) for FCN retraining. Finally, a 3D FCN with spatial context was employed (on all volumes; including the ones in the training set) to further improve the masks and experts provided final manual corrections to obtain accurate 3D reconstructions for quantitative analysis. Analyses of final 3D segmentations were performed at the Pennsylvania State University. Segmentation improvements (bottom): Subfigures 1(a)–(d) show improvements in segmentation masks (of the same specimen) through different stages of the pipeline. (a) With rep. slice annotations training data: 3/22 volumes, ~3%–4% slices annotated per volume. (b) With additional annotations training data: 1 additional volume, ~4% slices annotated in that volume. (c) With corrections + region specific annotations training data: 2 additional volumes, ~5% slices annotated per volume. (d) With spatial context from 3D FCN.

Our previous works (Perrine, Susan, Durham, et al., 2022; Perrine, Susan, Kathleen Pitirri, et al., 2022) used a deep learning‐based framework (Zheng et al., 2020) to progressively segment cartilages in high‐resolution 3D PTA‐enhanced microCT images using extremely sparse annotations. We used a similar framework and added additional operations to obtain high‐quality segmentations in three steps (Figure 1):

*Step 1. Hand annotation and iterative identification of target objects (pseudo‐labels)*. We start by selecting highly representative two‐dimensional (2D) image slices (3%–5% of slices) from 3D microCT volumes using Representative Annotation (Zheng et al., 2019), an unsupervised learning framework for image slice selection; the selected image slices both cover the diverse structures in the entire set of 2D slices and minimize redundant (repeated) information in the selected slices. Experts then fully labeled the selected image slices using Avizo 2021.2 (Thermo Fisher Scientific, Waltham, MA). We used these annotated slices to train a judiciously designed fully convolutional network (FCN) model for segmentation of 2D slices. The trained FCN model was then used to segment the unannotated 2D slices of all the microCT volumes. The objects in the 2D slices thus segmented are called pseudo‐labels. We estimated the reliability of the pseudo‐labels by computing their associated uncertainty maps, which quantify the pixel‐wise prediction (segmentation) confidence. Guided by the uncertainty maps and using the combined set of annotated slices and pseudo‐labels, the FCN model was iteratively trained to distill more stable knowledge about the training data and to enhance its robustness to unseen data. We attained the predictions for the objects of segmentation (called masks) along three orthogonal planes (*x*–*y*, *y*–*z*, *x*–*z*) of the microCT volumes and averaged them. These averaged prediction results are fine‐tuned in step 2.

*Step 2. Fine‐tuning with manual corrections*. We fine‐tuned this segmentation model with corrected labels because its generalizability is constrained by the highly sparse annotation and the unbalanced amounts of training pixels between easy and difficult regions. Due to this, the quality of segmentation after the first step on extremely difficult ROIs ([region of interest] e.g., MC and thinner parts of cranial vault cartilages) may still not meet the requirement of quantitative analysis (Figure 1a). Hence, in the second step, we evaluated the inadequately segmented regions with expert inspections and manually corrected the algorithm‐generated predictions. We combined the corrected annotations with the pseudo‐labels to further fine‐tune our segmentation model. Consequently, most specimens were segmented almost perfectly by our model, except for extremely thin, small, or variable, ambiguous regions in certain specimens outside our ability to capture at this resolution. Figure 1b, c show the cartilage predictions obtained from the different stages of fine‐tuning. The partial volume effect was not specifically considered in these thin regions since our model processed slices at a sufficiently high resolution.

*Step 3. Training a 3D FCN with contextual awareness using the results from the fine‐tuned 2D model*. To deal with possible segmentation errors in the troublesome regions and account for any inter‐slice artifacts resulted from the computation along the three orthogonal planes, we used the cartilage segmentations obtained by the 2D model after the fine‐tuning stage to train a 3D FCN model with contextual awareness to further improve segmentations. The 3D FCN removed isolated over‐segmentation artifacts, outputted more spatially coherent masks across neighboring slices, and qualitatively improved segmentation accuracy (Figure 1d). Experts manually validated the predictions and corrected any remaining errors in the segmentation results by the 3D FCN to generate the final chondrocranium segmentations for quantitative analysis.

### 2.5 Anatomical landmark acquisition

**FIGURE 2:**
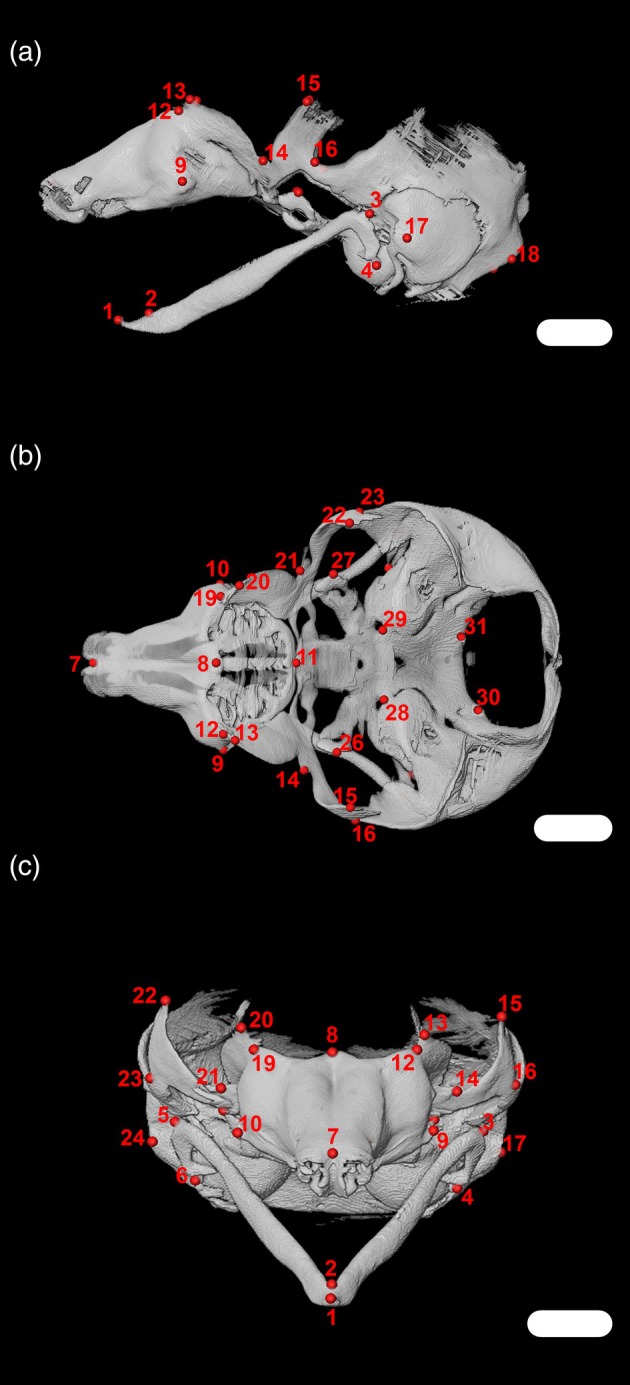
Anatomical landmark placement. Placement of anatomical landmarks on a 3D volumetric rendering of an E16.5 *Fgfr3* ^*C367Y/+*^ mouse chondrocranium. Landmarks are described in Table 1, and shown in left lateral (a), superior (b), and frontal (c) views. More information on landmark identification and location can be found at: http://getahead.la.psu.edu/landmarks. Scale bars = 1 mm.

Three‐dimensional coordinates of biologically relevant landmarks were collected from 2D slices and 3D isosurfaces created from PTA‐enhanced microCT images of the specimens using Avizo 2021.2 (ThermoFisher Scientific, Waltham, MA). Each specimen was digitized twice by the same observer, who was blinded to genotype. Percent difference (PD) in 3D landmark coordinate placement was calculated for each landmark as follows: PD = $\left(\frac{n2-n1}{n2}\right)\times 100$. A maximum of 5% error in landmark placement was considered acceptable. Landmarks with greater than 5% difference in placement were corrected, that is, landmarks were retaken for trias 1 and 2 and PD recalculated. This process was repeated, if necessary, until error was less than 5% PD for all landmarks. Intraobserver measurement error was further minimized by averaging the coordinates of the two trials. Table 1 provides anatomical definitions of all landmarks used; Figure 2 visualizes landmark position. Further information on landmark data can be found at https://getahead.la.psu.edu/landmarks/.

**TABLE 1:** Anatomical definitions of Meckel's cartilage (MC) and chondrocranial (C) landmarks used in morphological analyses and displayed in Figure 2.

Landmark number	Landmark abbreviation	Landmark definition	Tissue
1	mca	Most anterior point on the tip of Meckel's cartilage	MC
2	mcsym	Most posteriosuperior point on the symphysis of Meckel's cartilage	MC
3	mcpl	Most posterior point on the left arm of Meckel's cartilage	MC
4	mcppl	Most inferior point on the posterior aspect of the left arm of Meckel's cartilage	MC
5	mcpr	Most posterior point on the right arm of Meckel's cartilage	MC
6	mcppr	Most inferior point on the posterior aspect of the right arm of Meckel's cartilage	MC
7	asep	Most anterior point of the nasal septum	C
8	psep	Intersection of the superoposterior aspect of the nasal septum with the nasal capsule	C
9	lppi	Most lateral point on the prominent pars intermedia (nasal capsule), left side	C
10	rppi	Most lateral point on the prominent pars intermedia (nasal capsule), right side	C
11	nct	Most posterior midpoint at which the left and right nasal capsule connects with the trabecular cartilage	C
12	lncse	Most superior anterior point where the nasal capsule (pars intermedia) intersects with the sphenethmoid commissure (CSE), left side	C
13	lao	Most superolateral point on the ala orbitailis, left side	C
14	laottr	Most superior point of the intersection of the ala orbitailis and tectum transversum, left side	C
15	lttr	Most superior point on TTR (tectum transversum), left side	C
16	lttrpp	Most superior point on the intersection of the tectum transversum and the parietal plate, left side	C
17	llpca	Most lateral point on the pars canalicularis, left side	C
18	ltpoa	Intersection of the tectum posterius (TP) and occipital arch (OA) on the foramen magnum, left side	C
19	rncse	Most superior anterior point where the nasal capsule (pars intermedia) intersects with the sphenethmoid commissure (CSE), right side	C
20	rao	Most uperolateral point on the ala orbitailis, right side	C
21	raottr	Most superior point of hte intersection of the ala orbitailis and tectum transversum, right side	C
22	rttr	Most superior point on TTR (tectum transversum), right side	C
23	rttrpp	Most superior point on the intersection of the tectum transversum and the parietal plate, right side	C
24	rlpca	Most lateral point on the pars canalicularis, right side	C
25	rtpoa	Intersection of the tectum posterius (TP) and occipital arch (OA) on the foramen magnum, right side	C
26	llat	Most lateral point on the ala temporalis, left side	C
27	rlat	Most lateral point on the ala temporalis, right side	C
28	lcsp	Intersection of the sphenocochlear commissure (CSC) and pars cochlearis (PCO), left side	C
29	rcsp	Intersection of the sphenocochlear commissure (CSC) and pars cochlearis (PCO), right side	C
30	laioc	Most posterior inferior point on the occipital condyle, left side	C
31	raioc	Most posterior inferior point on the occipital condyle, right side	C

### 2.6 Statistical analysis of data

#### 2.6.1 Morphological comparison and growth of embryonic cartilage

Euclidean distance matrix analysis (EDMA) was used to statistically determine morphological differences between groups (Lele, 1993; Lele & Richtsmeier, 1995, 2001). EDMA converts 3D landmark data into a matrix of all possible unique linear distances (the form matrix) for statistical testing and ratios of like linear distances are used to estimate the relative changes in geometry between genotypes at each age. We used groups of landmarks representing the chondrocranium and MC to statistically test for anatomical differences between *Fgfr3* ^*Y367C/+*^ embryos and their *Fgfr3* ^*+/+*^ littermates at E14.5 and E16.5. Statistical significance for specific linear distances is evaluated by confidence intervals (α ≤ 0.10) produced through a non‐parametric bootstrapping procedure enabling localization of differences to specific dimensions. All EDMA analyses were performed using both WinEDMA (University of Missouri‐Kansas City, MO, USA; Cole III, 2002) and EDMAinR (University of Alberta, Edmonton, Canada; Solymos, 2021) to allow a complete exploration of the data as different viewing features are available in each package. Reported results were obtained using WinEDMA.

To statistically compare growth from E14.5 to E16.5 across genotypes, we used the growth difference matrix analysis (GDMA) module of EDMA (Lele & Richtsmeier, 2001; Richtsmeier & Lele, 1993). 3D landmark data for each age group within genotypes are first converted into a matrix of all possible unique linear distances (the form matrix) for statistical testing. We used groups of landmarks representing the chondrocranium and MC to statistically test for differences in growth from E14.5 to E16.5 between *Fgfr3* ^*Y367C/+*^ embryos and their *Fgfr3* ^*+/+*^ littermates. To estimate growth for each group, the form matrix of the older (E16.5) mouse serves as the numerator, and the form matrix of the younger (E14.5) mouse serves as the denominator, and ratios are estimated elementwise, resulting in the growth matrix (GM). The GM estimates growth for each genotype as the relative change in the lengths of all unique linear distances between landmarks. A growth difference matrix (GDM) compares the growth of the two samples using the growth matrices through an element‐wise comparison of growth for each linear distance. Elements of the GDM are statistically evaluated using methods similar to those described above form EDMA using non‐parametric bootstrapping and confidence interval testing (α = 0.10) (Lele & Richtsmeier, 2001; Richtsmeier & Lele, 1993).

#### 2.6.2 Principal components analysis of form and shape

Ontogenetic variation in chondrocranium and MC form and shape was assessed using principal component analysis (PCA), an approach that summarizes the variation of many variables into a lower‐dimensional space defined by principal component (PC) axes that are mutually orthogonal, linear combinations of the linear distance data. The scores of an observation along the PC axes map that observation into space. We performed PCA (Darroch & Mosimann, 1985; Falsetti et al., 1993) of form (size and shape together) using interlandmark linear distances estimated using either the full chondrocranium landmark set or the full MC landmark set. Interlandmark distances were *ln*‐transformed, and their variance–covariance matrix was used as the basis for the PCA. For shape alone, the linear measures were used to define dimensionless shape variables, where all information about the absolute size of the measurements was removed by scaling by the geometric mean, and only information about proportions remained. The amount of variation due to form space (size and shape) is the sum of variances for all *ln*‐transformed linear measurements, while the amount of variance due to shape alone (shape space) is the sum of variances for the *ln*‐transformed ratios. The difference in these is the amount of variance due to size alone (Perrine et al., 2014). The PCAs were performed in SAS 9.4 (SAS Institute, Cary, NC, USA) as described in (Perrine et al., 2014).

### 2.7 Cartilage thickness

Average object thickness was determined using the parallel plate model measuring thickness of 3D image structure in Avizo 2021.2. Thickness is defined by this model as:

$$\text{Average Object Thickness}=2/\left(\frac{\mathrm{Obj}*S}{\left(\mathrm{Obj}*V\right)}\right),$$

where Obj*S/Obj*V is the surface‐to‐volume ratio (sr.th). The image input was interpreted as 3D volumes for processing. Cartilage volumes were compared between genotypes at each age by nonparametric Mann–Whitney U tests in IBM SPSS 25 (IBM, Armock, NY) as there were violations of assumptions of homogeneity of variance and/or normality.

Visualizations for representative specimens were mapped using the Thickness Map module of Avizo 2021.2. This module computes a voxel‐wise thickness for 3D objects (Hildebrand & Rüegsegger, 1997). The thickness value for each voxel represents the diameter of the largest ball containing the voxel and was entirely inscribed in the object. The algorithm started by computing a Euclidean distance map inside the image objects. In a second step, the thickness *T* was initialized with the values of the distance map. For each voxel *v*, with distance map value *d*(*v*), the ball *B* centered on *v* and with radius *R = d*(*v*) was browsed. The thickness of *T* of these neighboring voxels *w* was set to *max*(*T*(*w*)*R*). Finally, the thickness map is multiplied by 2 to obtain the largest ball diameter. Boundary voxels were determined based upon the averages of two distance maps. The first distance map, termed excluded, computed the distance map inside the object, starting from the boundary voxels belonging to the object. This option tends to slightly reduce the thickness measured on the boundary voxels of the structure. The second distance map, termed included, computed the distance map inside the object, starting from the voxels center to just outside the object of interest. This option tends to slightly increase the thickness measured on the boundary voxels of the structure. Therefore, the averaged option was chosen, which averaged the distance between the included and excluded distance maps for a smoother result at the expense of increased computation time. The minimum (left point) of the user‐defined colormap was set to blue to indicate thin cartilage, and the maximum (right point) of the colormap was set to red to indicate thicker cartilage. The same colormap scale used included all values obtained among all mapped specimens.

### 2.8 Curve measurement

Total MC length along the superior surface of the 3D surface model reconstructions of MC was measured using the open curve tool (spline curve type, constrained to model) of the Markups model in 3D Slicer software (Fedorov et al., 2012). Circumference of the foramen magnum was measured on the 3D surface model reconstructions of chondrocrania using the closed curve tool (spline curve type, constrained to model) of the Markups module in 3D Slicer software (Fedorov et al., 2012) on 3D reconstructions of the chondrocrania at E14.5 and E16.5.

## 3 RESULTS

### 3.1 Morphological differences of the chondrocranium

**FIGURE 3:**
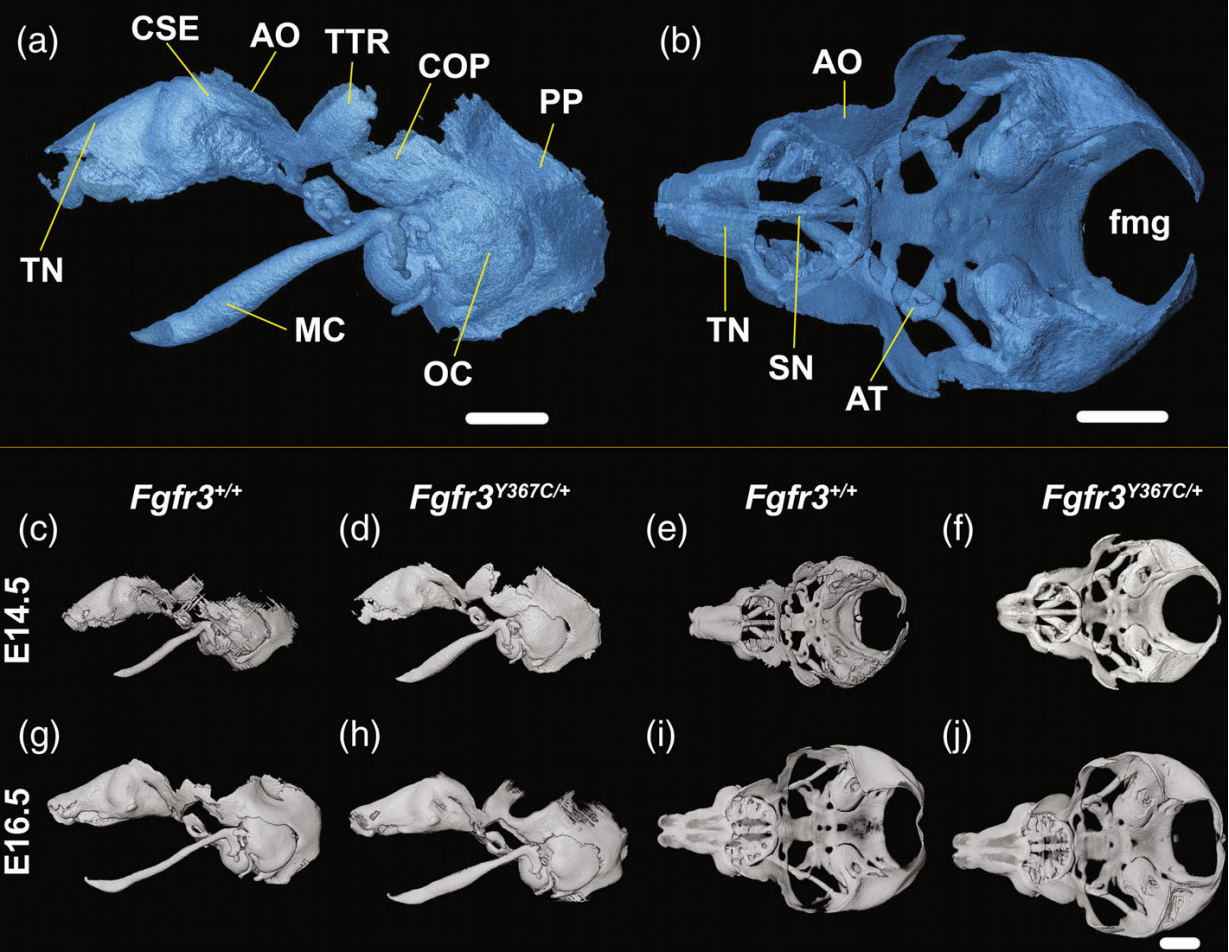
Anatomy of embryonic mouse chondrocrania and Meckel's cartilage. Three‐dimensional (3D) reconstruction of an *Fgfr3* ^*C367Y/+*^ mouse embryonic chondrocranium at E16.5 segmented from phosphotungstic acid (PTA) enhanced micro‐computed tomography (microCT) volumes in lateral (a) and superior (b) views, depicting specific anatomical areas of interest, including the ala orbitalis (AO), ala temporalis (AT), orbitoparietal commissure (COP), sphenethmoid commissure (CSE), Meckel's cartilage (MC), otic capsule (OC), parietal plate (PP), septum nasi (SN), and tectum transversum (TTR) cartilages, and the foramen magnum (fmg). 3D reconstructions of *Fgfr3* ^*+/+*^ (c), (e) and *Fgfr3* ^*C367Y/+*^ (d), (f) chondrocrania and Meckel's cartilage at E14.5 in lateral (c), (d) and superior views (e), (f). 3D reconstructions of *Fgfr3* ^*+/+*^(g), (i) and *Fgfr3* ^*C367Y/+*^ (h), (j) chondrocrania and Meckel's cartilage at E16.5 in lateral (g), (h) and superior views (i), (j). All scale bars = 1 mm.

3D volumes of chondrocrania at E14.5 and E16.5, segmented from PTA‐enhanced microCT images of *Fgfr3* ^*+/+*^ and *Fgfr3* ^*Y367C/+*^ mice, show gross morphological differences between the genotypes and across ages (Figure 3; Video 1). To quantify the differences, a set of 25 anatomical landmarks was used to describe the global shape of the chondrocranium at E14.5 and E16.5 (Table 1; Figure 2).

**VIDEO 1:** Anatomy of embryonic *Fgfr3* ^*C367Y/+*^ (left) and *Fgfr3* ^*+/+*^ (right) mouse chondrocrania and Meckel's cartilage at E14.5 (top row) and E16.5 (bottom row). Three‐dimensional (3D) volume renderings of *Fgfr3* ^*C367Y/+*^ mouse embryonic chondrocrania at E14.5 (top left) and E16.5 (bottom left) and *Fgfr3* ^*+/+*^ mouse chondrocrania at E14.5 (top right) and E16.5 (bottom right) segmented from phosphotungstic acid (PTA) enhanced micro‐computed tomography (microCT) volumes in lateral view. Scale bars = 1 mm.

**FIGURE 4:**
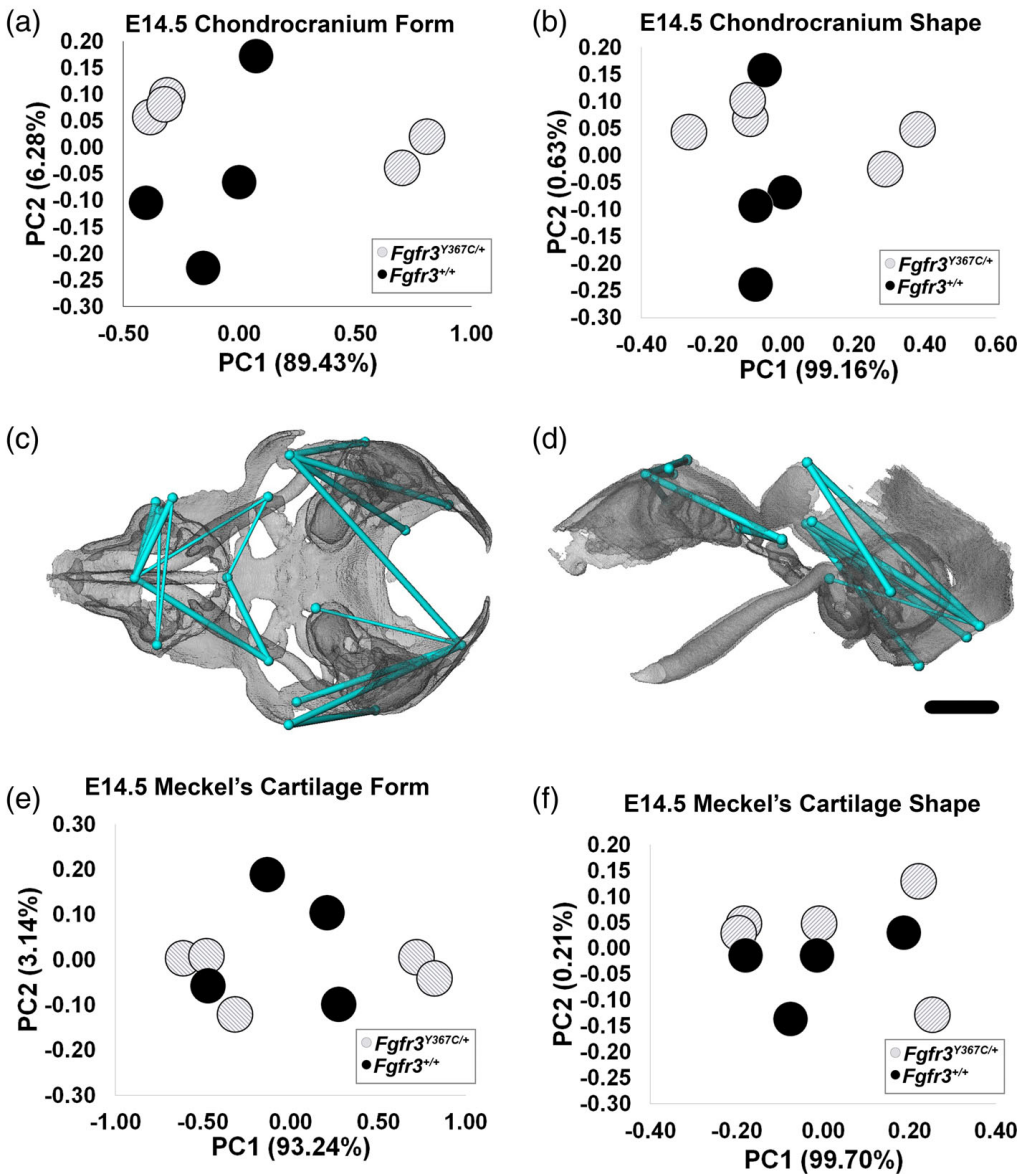
Geomorphometric analysis of the chondrocranium and Meckel's cartilage at E14.5. Principal components analysis (PCA) of form and shape for the chondrocranium (a), (b) and Meckel's cartilage (e), (f) at E14.5. Linear distances estimated between 3D landmark locations on the chondrocranium and Meckel's cartilage that are statistically different between genotypes by confidence interval testing at E14.5 (c), (d) in superior (c) and left lateral (d) views. These significant differences in linear distances are shown on *Fgfr3* ^*C367Y/+*^ chondrocrania at E14.5. Blue lines indicate linear distances that are significantly larger in *Fgfr3* ^*C367Y/+*^ mice as compared to *Fgfr3* ^*+/+*^ littermates. 3D reconstructions of E14.5 chondrocrania segmented from PTA enhanced microCT of *Fgfr3* ^*Y367C/+*^ embryos were used for visualization. Scale bar = 1 mm.

*E14.5* PCA of all 300 unique inter‐landmark distances estimated from 25 global chondrocranium landmarks was used as an exploratory step in describing and comparing chondrocranial morphologies. A plot of the first two principal components of the E14.5 chondrocranial data reveals that chondrocranial morphologies of *Fgfr3* ^*+/+*^ and *Fgfr3* ^*Y367C/+*^ mice overlap along the first and second principal component axes (PC1 and PC2) in chondrocranial form space (Figure 4a; E14.5 Chondrocranium Form). The mice plotting to the positive aspect of PC1 are shorter from the most anterior point of the nasal capsule to the most posterior aspect of the cranial base area of the chondrocranium describing the foramen magnum. These specimens also have higher lateral walls of the chondrocranium, specifically in the tectum transversum (TTR, Figure 3a) as compared to those plotting on the negative aspect of PC1. Specimens plotting along the positive axis of PC2 show a more posterior placed tip of the TTR, while specimens plotting along the negative axis of PC2 show a more anterior‐placed tip of the TTR. Placement of all E14.5 mouse chondrocrania on PC1 and PC2 in the chondrocranial shape space (as estimated by PCA of all possible linear distances of each observation, scaled by the observation's geometric mean) also showed overlap along PC1 and PC2 (Figure 4b; chondrocranium shape). Specimens plotting along the positive axis of PC1 show a decreased height of lateral walls of the chondrocranium, specifically in the height of the TTR, along with a wider posterior nasal capsule and shorter cranial base as determined by the landmarks describing the inferior and lateral points of the foramen magnum, while those on the negative axis of PC1 display taller lateral TTR and elongated most posterior aspect of the cranial base and occipital walls. Specimens plotting along the positive axis of PC2 display a taller, more posteriorly placed TTR as compared to those on the negative axis.

Patterns of 3D shape differences between *Fgfr3* ^*+/+*^ and *Fgfr3* ^*Y367C/+*^ mice were evaluated using EDMA. Many aspects of the chondrocrania of *Fgfr3* ^*Y367C/+*^ mice were larger compared to *Fgfr3* ^*+/+*^ littermates. Localized differences were discovered through confidence interval testing of these inter‐landmark distances at E14.5 and revealed five linear distances that were 5%–10% larger and 12 linear distances that were 11%–30% larger in *Fgfr3* ^*Y367C/+*^ embryos as compared to *Fgfr3* ^*+/+*^ littermates. The affected dimensions describe the dorsal aspect of the nasal capsule, the medial portion of the cranial base rostral to the otic capsules, and the length of the lateral walls, preoccipital, and occipital regions (Figure 4c, d; Video 2). All linear distances describing the lateral walls were 11%–30% larger in *Fgfr3* ^*Y367C/+*^ mice relative to *Fgfr3* ^*+/+*^ littermates.

**VIDEO 2:** Geomorphometric analysis of the *Fgfr3* ^*C367Y/+*^and *Fgfr3* ^*+/+*^ chondrocrania and Meckel's cartilage at E14.5. Significant differences in linear distances as determined through confidence interval testing of Euclidean distance matrix analysis (EDMA) are shown on a three‐dimensional (3D) volume rendering of a *Fgfr3* ^*C367Y/+*^ chondrocranium and Meckel's cartilage at E14.5 segmented from phosphotungstic acid (PTA) enhanced microcomputed tomography (microCT) images. Blue lines indicate linear distances that are at least 5% significantly larger using α = 0.10 confidence limits in *Fgfr3* ^*C367Y/+*^ mice as compared to *Fgfr3* ^*+/+*^ littermates at E14.5. Scale bar = 1 mm.

**FIGURE 5:**
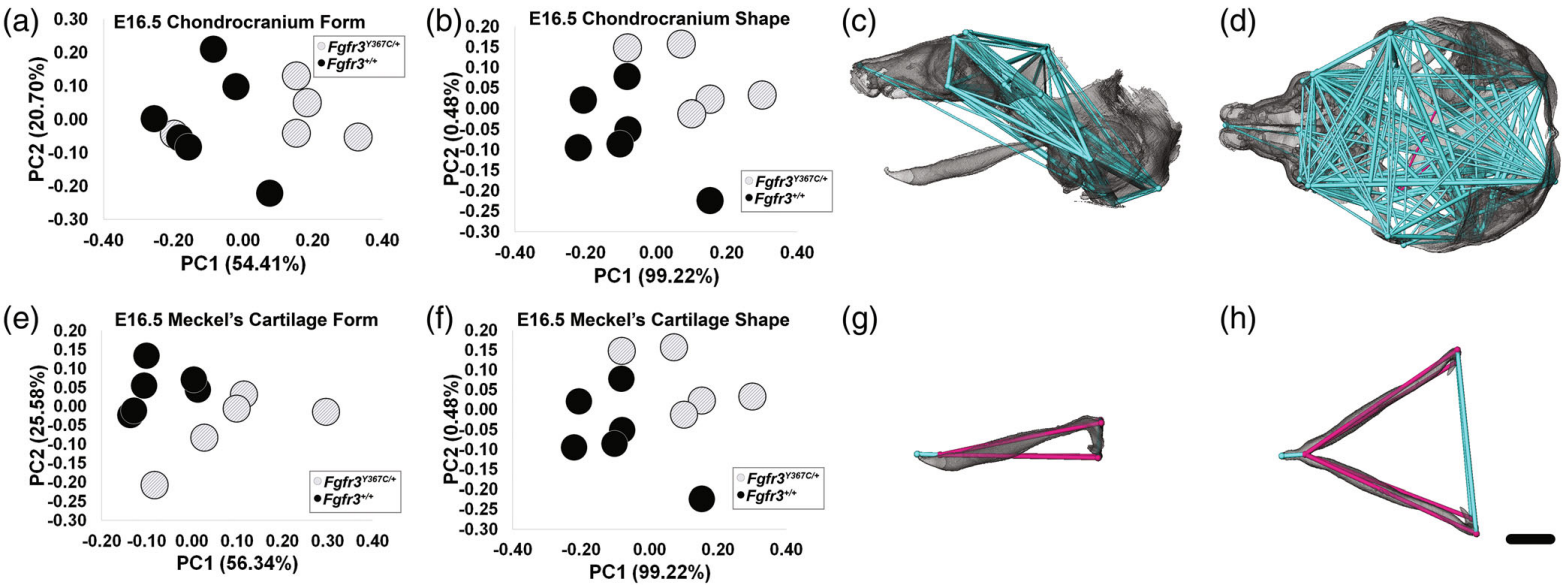
Geomorphometric analysis of the chondrocranium and Meckel's cartilage at E16.5. Principal components analysis (PCA) of form and shape for the chondrocranium (a), (b) and Meckel's cartilage (g), (e) at E16.5. Linear distances estimated between 3D landmark locations on the chondrocranium (c), (d) and Meckel's cartilage (g), (h) that are statistically different between genotypes by confidence interval testing at E16.5 in left lateral (c), (g) and superior (d), (h) views. These significant differences in linear distances are shown on *Fgfr3* ^*C367Y/+*^ chondrocrania at E16.5. Blue lines indicate linear distances that are significantly larger in *Fgfr3* ^*C367Y/+*^ mice as compared to *Fgfr3* ^*+/+*^ littermates, while pink lines indicate linear distances that are significantly reduced in *Fgfr3* ^*C367Y/+*^ mice. 3D reconstructions of E16.5 chondrocrania segmented from PTA enhanced microCT of *Fgfr3* ^*Y367C/+*^ embryos were used for visualization. Scale bar = 1 mm.

*E16.5* PCA of 300 unique inter‐landmark distances estimated from 25 global chondrocranium landmarks was used as an exploratory step in describing and comparing chondrocranial morphologies. A plot of the first two principal components of the E16.5 chondrocranial data reveals that chondrocranial morphologies of most *Fgfr3* ^*+/+*^ and *Fgfr3* ^*Y367C/+*^ mice separate well along PC1 but overlap along PC2 in chondrocranial form space (Figure 5a; E16.5 Chondrocranium Form). Specimens plotting along the positive axis of PC1 have taller lateral walls of the chondrocranium, with an overall wider cranial vault and reduced width of the cranial base. Specimens plotting along the positive aspect of PC2 have a reduced height of the lateral chondrocranial walls (specifically in the area of the TTR and preoccipital lateral walls, with an increased height to the most posterior aspect of the occipital lateral walls. The separation along PC1 may be due to size differences between the *Fgfr3* ^*+/+*^ and *Fgfr3* ^*Y367C/+*^ mice. One *Fgfr3* ^*Y367C/+*^ specimen plots near the middle of the grouping of *Fgfr3* ^*+/+*^ specimens, while the remaining *Fgfr3* ^*Y367C/+*^ specimens group together. Results in the shape space, as estimated by PCA of all possible linear distances of each observation, scaled by the observation's geometric mean, showed some separation of the groups along both PC1 and PC2 (Figure 5b; chondrocranium shape). Shape variation along PC1 was primarily associated with the height of the lateral chondrocranial walls. Specimens plotting along the positive aspect of PC1 showed increased height of the TTR. Shape variation along the positive aspect of PC2 was primarily associated with shorter lateral walls of the chondrocranium, increased height of the most posterior aspect of the occipital lateral walls, and increased cranial vault and cranial base widths.

We used EDMA to statistically determine localized changes in morphology of the chondrocranium at E16.5. Confidence interval testing revealed many significant localized differences. At E16.5, 136 linear distances were of significantly increased length in *Fgfr3* ^*Y367C/+*^ mice relative to *Fgfr3* ^*+/+*^ littermates, including 84 linear distances that were increased in length by 5%–10% and 53 linear distances increased in length by 11%–41% (Figure 5c, d; Video 3) indicating overall larger chondrocrania in *Fgfr3* ^*Y367C/+*^ mice. Most linear distances that differed by a magnitude of 11% or greater in *Fgfr3* ^*Y367C/+*^ embryos as compared to *Fgfr3* ^*+/+*^ embryos at E16.5 were found in the lateral walls of the chondrocranium, specifically the area of the ala orbitalis (AO; Figure 5c, d), TTR, and measures stretching from the TTR to the ala temporalis and the nasal capsule. Chondrocranial width is also significantly increased in *Fgfr3* ^*Y367C/+*^ embryos at E16.5. Three linear distances describing the foramen magnum and two describing the mid portion of the cranial base between the intersection of the sphenocochlear commissure (CSC) and the pars cochlearis (PCO) and most lateral points on the ala temporalis were reduced in length in *Fgfr3* ^*Y367C/+*^ embryos relative to *Fgfr3* ^*+/+*^ embryos at E16.5.

**VIDEO 3:** Geomorphometric analysis of the *Fgfr3* ^*C367Y/+*^and *Fgfr3* ^*+/+*^ chondrocrania and Meckel's cartilage at E16.5. Significant differences in linear distances as determined through confidence interval testing of Euclidean distance matrix analysis (EDMA) are shown on a three‐dimensional (3D) volume rendering of a *Fgfr3* ^*C367Y/+*^ chondrocranium and Meckel's cartilage at E16.5 segmented from phosphotungstic acid (PTA) enhanced microcomputed tomography (microCT) images. Blue lines indicate linear distances that are significantly larger in *Fgfr3* ^*C367Y/+*^ mice as compared to *Fgfr3* ^*+/+*^ littermates, whereas pink lines indicate linear distances that are significantly shorter in *Fgfr3* ^*C367Y/+*^ mice as compared to *Fgfr3* ^*+/+*^ littermates at E16.5. The linear distances pictured are limited to those that differed between genotypes by at least 5% using α = 0.10 confidence limits. Scale bar = 1 mm.

### 3.2 Morphological differences of MC

3D volumes of MC at E14.5 and E16.5, segmented from PTA‐enhanced microCT images of *Fgfr3* ^*+/+*^ and *Fgfr3* ^*Y367C/+*^ mice, show gross morphological differences between the genotypes and across ages (Figure 3; Video 1). To quantify morphological differences, a set of six anatomical landmarks was used to describe the global shape of MC at E14.5 and E16.5 (Table 1; Figure 2).

*E14.5* PCA of form at E14.5 was performed using the 15 unique inter‐landmark linear distances describing Meckel's cartilage. There was overlap of groups along both PC1 and PC2 (Figure 4g; MC Form). Individuals also overlapped following scaling by each individual's geometric mean. Data were plotted within the shape space resulting in overlap of groups along both PC1 and PC2 (Figure 4e; MC shape). At E14.5, there are no statistically significant localized differences in linear distances of MC as determined by EDMA confidence interval testing.

*E16.5* PCA of form at E16.5 revealed partial separation of the groups along PC1 and overlap along PC2 (Figure 5e; E16.5 MC form). When size was accounted for and data were plotted in the MC shape space, there was a separation between the groups at E16.5 along PC1, indicating shape differences (Figure 5f; E16.5 MC shape). Specific localized differences at E16.5, deduced from confidence interval testing, include a 3%–5% relative decrease in rostrocaudal length of MC rods as described as the linear distance between the most posterior point on the symphysis of MC to the two most posterior points on the bilateral rods of MC in *Fgfr3* ^*Y367C/+*^ embryos. The length of the MC symphysis is approximately 12% longer in *Fgfr3* ^*Y367C/+*^ embryos as compared to *Fgfr3* ^*+/+*^ embryos (Figure 5g, h; Video 3). Linear distances associated with the four landmarks of the proximal ends of MC are 3%–8% longer in *Fgfr3* ^*Y367C/+*^ embryos as compared to *Fgfr3* ^*+/+*^ littermates at E16.5, implying a posteriorly wider MC in *Fgfr3* ^*Y367C/+*^ embryos.

### 3.3 Growth‐related shape variation of the chondrocranium

**FIGURE 6:**
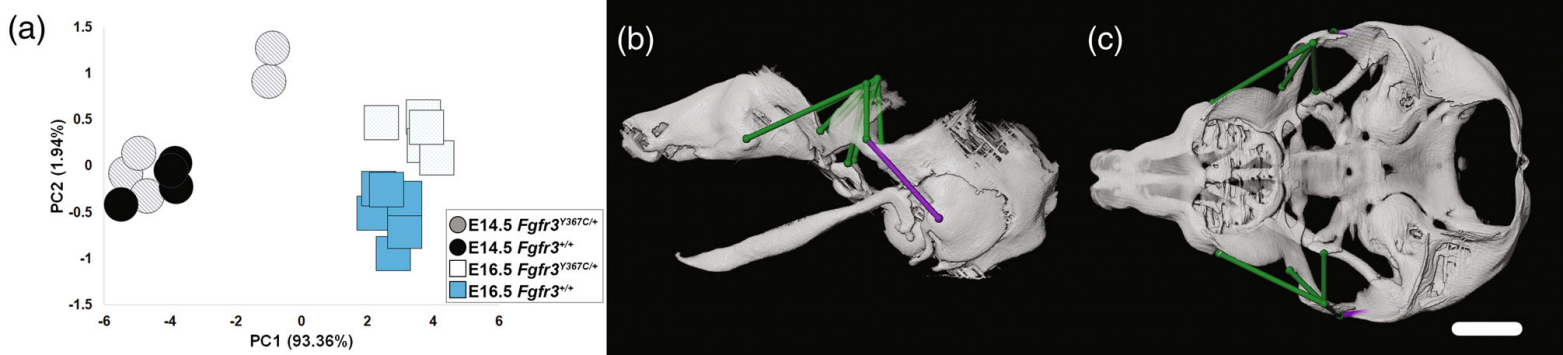
Geomorphometric analysis of growth of the chondrocrania at E14.5 and E16.5. Placement of E14.5 and E16.5 mouse chondrocrania on PC1 and PC2 in the chondrocranial form space as estimated by principal component analysis of all linear distances among 25 chondrocranial landmarks (a). Differences in growth between *Fgfr3* ^*Y367C/+*^ mice as compared to *Fgfr3* ^*+/+*^ littermates were determined by growth difference matrix analysis (GDMA). Differences in growth between E14.5 and E16.5 *Fgfr3* ^*Y367C/+*^ mice as compared to *Fgfr3* ^*+/+*^ littermates in left lateral (b) and superior (c) views. Growth differences are shown on an E16.5 *Fgfr3* ^*Y367C/+*^ chondrocranium. The linear distances pictured are limited to those in which growth of the chondrocranium from E14.5 to E16.5 differed significantly by at least 5% using α = 0.10 confidence limits. Linear distances that grew significantly more (green) and significantly less (purple) in *Fgfr3* ^*Y367C/+*^ mice as compared to *Fgfr3* ^*+/+*^ littermates from E14.5 to E16.5 are shown. All significant growth differences involve the lateral walls of the chondrocranium. Scale bar = 1 mm.

Principal components analysis of ontogenetic variation revealed that chondrocranial morphology was similar in the two groups at E14.5 but much different by E16.5 (Figure 6a). PC1 primarily reveals the impact of developmental age, with the chondrocrania of E14.5 specimens clustering toward the negative end of PC1 and the older E16.5 specimens situated at the positive end (Figure 6a). PC2 separates groups according to genotype, although the groups overlap at E14.5. GDMA was performed to further elucidate differences in growth from E14.5 and E16.5 between *Fgfr3* ^*Y367C/+*^ mice and *Fgfr3* ^*+/+*^ littermates. The caudal portion of the nasal capsule and preoccipital portion of the lateral walls rostral to the orbitoparietal commissure (COP, Figure 3a) grew significantly more (7.5%–17.7%) in *Fgfr3* ^*Y367C/+*^ embryos relative to *Fgfr3* ^*+/+*^ littermates from E14.5 to E16.5, although there was more than a 20% reduction in lateral wall growth of *Fgfr3* ^*Y367C/+*^ embryos from the COP to the otic capsules (Figure 6b, c; Video 4).

**VIDEO 4:** Geomorphometric analysis of growth of the chondrocrania at E14.5 and E16.5. Differences in growth between *Fgfr3* ^*Y367C/+*^ mice as compared to *Fgfr3* ^*+/+*^ littermates were determined by Growth difference matrix analysis (GDMA). Differences in growth between E14.5 and E16.5 *Fgfr3* ^*Y367C/+*^ mice as compared to *Fgfr3* ^*+/+*^ littermates are shown on a three‐dimensional (3D) volume rendering of the chondrocranium and Meckel's cartilage of an E16.5 5 *Fgfr3* ^*Y367C/+*^ mouse segmented from phosphotungstic acid (PTA) enhanced microcomputed tomography (microCT) images. The linear distances pictured are limited to those in which growth of the chondrocranium from E14.5 to E16.5 differed significantly by at least 5% using α = 0.10 confidence limits. Linear distances that grew significantly more (green) and significantly less (purple) in *Fgfr3* ^*Y367C/+*^ mice as compared to *Fgfr3* ^*+/+*^ littermates from E14.5 to E16.5 are shown. Scale bar = 1 mm.

### 3.4 Cartilage thickness and foramen magnum size in *Fgfr3* ^*Y367C*^ ^*/+*^ as compared to *Fgfr3* ^*+/+*^ embryos

**FIGURE 7:**
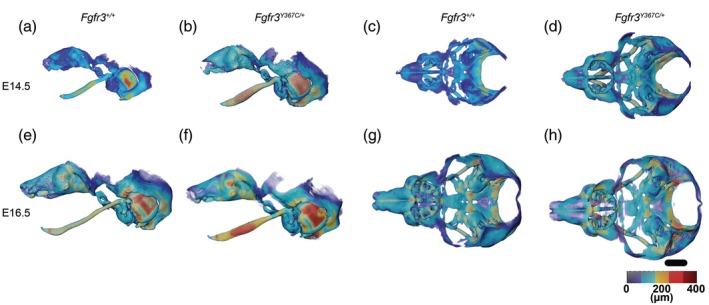
Thickness maps of the chondrocranium of E14.5 and E16.5 *Fgfr3* ^*C367Y/+*^ and *Fgfr3* ^*+/+*^ mice segmented from PTA‐enhanced microCT volumes. Thickness maps of E14.5 *Fgfr3* ^*+/+*^ (a), (c) and *Fgfr3* ^*C367Y/+*^ (b), (d) mice in left lateral (a), (b) and superior views (c), (d). Thickness maps of E16.5 *Fgfr3* ^*+/+*^ (e), (g) and *Fgfr3* ^*C367Y/+*^ (f), (h) mice in left lateral (e), (f) and superior views (g), (h). Colormap bar indicates cartilage thickness ranged from just over 0 μm to nearly 400 μm. Scale bar = 1 mm.

The thickness of chondrocrania was estimated using the Average Object Thickness algorithm in Avizo 2021.2 and was generally less in E14.5 as compared to E16.5 specimens of both genotypes and was similar between *Fgfr3* ^*Y367C/+*^ and *Fgfr3* ^*+/+*^ embryos at E14.5 (*p* = 0.556), but markedly thicker in the *Fgfr3* ^*Y367C/+*^ at E16.5 (*p* = 0.009; Table 2, Figure 7). MC was thicker in *Fgfr3* ^*Y367C/+*^ as compared to *Fgfr3* ^*+/+*^ embryos at E14.5 (*p* = 0.016) and E16.5 (*p* = 0.004) (Table 2, Figure 7). The colormap for chondrocranial and MC thickness indicates regions of lesser and greater cartilage thickness.

**TABLE 2:** Cartilage thickness in *Fgfr3* ^*Y367C/+*^ and *Fgfr3* ^*+/+*^ mice at E14.5 and E16.5.

Age	E14.5			E16.5		
Genotype	*Fgfr3* ^*Y367C/+*^	*Fgfr3* ^*+/+*^		*Fgfr3* ^*Y367C/+*^	*Fgfr3* ^*+/+*^	
*n*	*5*	*4*	*p*‐value	*5*	*6*	*p*‐value
Chondrocranial Thickness (Sr.th)	0.083 ± 0.004	0.059 ± 0.004	0.556	0.128 ± 0.003	0.112 ± 0.002	**0.009**
Meckel's Cartilage Thickness (Sr.th)	0.105 ± 0.005	0.077 ± 0.002	**0.016**	0.141 ± 0.001	0.102 ± 0.001	**0.004**
Total Meckel's Cartilage length as measured on the curve (mm)	5.93 ± 0.48	5.90 ± 0.27	0.905	9.77 ± 0.16	10.12 ± 0.09	0.126
Foramen Magnum Circumference (mm)	6.07 ± 0.12	5.81 ± 0.14	0.111	6.96 ± 0.12	7.96 ± 0.38	0.329

*Note*: Bold values represents α = 0.05.

Patterns of cartilage thickness (Figure 7) differed between the genotypes at both ages studied. Localized thickness is displayed as a thickness map (as described in Section 2.7) over the chondrocranium and MC. The colormap shown in the legend was applied to indicate areas of thinner cartilage, which plot on the left side of the color spectrum, as blue, and areas of thicker cartilage, which plot on the right side of the color spectrum, as red. At E14.5 more, thinner cartilage was formed in the preoccipital and occipital regions of the lateral walls in *Fgfr3* ^*Y367C/+*^ embryos as compared to *Fgfr3* ^*+/+*^ littermates. The otic capsules and medial and caudal aspects of the cranial base are generally thinner in *Fgfr3* ^*Y367C/+*^ embryos as compared to *Fgfr3* ^*+/+*^ littermates. The lateral edges of the most prominent portion of pars intermedia (nasal capsule), small areas of cartilage of the ethmoid cartilage, otic capsule walls, and most caudal aspect of the cranial base associated with the foramen magnum are thicker in *Fgfr3* ^*Y367C/+*^ embryos as compared to *Fgfr3* ^*+/+*^ littermates.

Foramen magnum circumference was estimated as the closed circle length on the 3D chondrocranial volumes (Table 2). At E14.5, there was no difference between the foramen magnum circumference in *Fgfr3* ^*Y367C/+*^ and *Fgfr3* ^*+/+*^ mice (*p* = 0.111). By E16.5, the foramen magnum circumference was significantly less in *Fgfr3* ^*Y367C/+*^ than in *Fgfr3* ^*+/+*^ mice (*p* = 0.017).

## 4 DISCUSSION

While it has been suggested that localized alteration of chondrocranial development contributes to distinct patterns of respiratory difficulty in young children with ACH (Tasker et al., 1998), this is the first study to quantitatively define the chondrocranium and MC during embryonic development of the *Fgfr3* ^*Y367C/+*^ mouse using PTA enhanced microCT imaging and novel machine learning methods of cartilage segmentation. Analysis of chondrocranial and MC forms reveals global and local variation in cartilage morphology and growth in mice carrying an *Fgfr3* variant associated with ACH in humans. At E14.5, all linear distances that are significantly different between genotypes (as shown in Figure 4c, d; Video 2) were longer in *Fgfr3* ^*Y367C/+*^ embryos relative to *Fgfr3* ^*+/+*^littermates. EDMA analyses indicated overall larger chondrocrania in *Fgfr3* ^*Y367C/+*^ embryos relative to *Fgfr3* ^*+/+*^littermates and determined localized differences of the posterior aspect of the nasal capsule, anterior portion of the cranial base, and lateral length and height of the lateral chondrocranial walls from the TTR to the most posterior aspects of the occipital lateral walls and cranial base. Our analyses revealed an increased height and length of the chondrocranial lateral walls in *Fgfr3* ^*Y367C/+*^ embryos relative to *Fgfr3* ^*+/+*^littermates at E16.5 in addition to increased length of the chondrocranial areas encasing the brain, along with a decreased inferior width of the foramen magnum (Figure 5). While some asymmetry was noted in the lateral walls of the chondrocrania and can be visualized in the representative specimens used for visualizations in Videos 2, 3, this biological variation could not be well quantified with the specimen numbers available. To date, we found no results in the literature (http://pubmed.ncbi.nlm.nih.gov) when “asymmetry,” “achondroplasia,” and “mouse” or “asymmetry,” “achondroplasia,” and “chondrocranium” were searched. The investigation of asymmetry of cartilage and bone growth in this mouse model could be a future endeavor.

GDMA indicated increased growth of the superior aspect of the preoccipital and occipital lateral walls encompassing the area of the AO and TTR from E14.5 and E16.5 in *Fgfr3* ^*Y367C/+*^ embryos relative to *Fgfr3* ^*+/+*^ littermates. The thickness of the chondrocranium at E14.5 was similar among the genotypes, but markedly thicker in *Fgfr3* ^*Y367C/+*^ embryos by E16.5. While the foramen magnum circumference was similar at E14.5, there was a reduction in circumference in *Fgfr3* ^*Y367C/+*^ embryos by E16.5. A similar phenotype of foramen magnum compression has been documented in children under 18‐year‐old with a confirmed diagnosis of ACH (Bosemani et al., 2015). Severe foramen magnum stenosis with cervicomedullary compression can increase the risk of sudden death if not diagnosed and treated appropriately (Savarirayan et al., 2022).

The chondrocranium is a complex structure that develops early in embryonic development and influences skull growth in bony vertebrates yet is transient in nature and thus difficult to characterize in 3D across developmental time. Initiation of chondrocranial growth begins with development of the parachordal cartilages at E12.5 in C57BL/6J mice (Kawasaki & Richtsmeier, 2017). Growth continues with lateral walls of the preoccipital region consisting of the AO, sphenethmoid commissure (CSE), and TTR by E14.5 (Kawasaki & Richtsmeier, 2017; Pitirri et al., 2020). Most elements of the chondrocranium and MC develop by E15.5, and portions begin to degrade by E16.5 as cranial dermal bones mineralize and grow (Kawasaki & Richtsmeier, 2017; Pitirri et al., 2020; Pitirri et al., 2022). Our results demonstrate that the region between the nasal capsule and the COP lateral walls grows relatively more, while the area of the lateral walls between the TTR and the otic capsules grows relatively less from E14.5 to E16.5 in mice carrying an *Fgfr3* Y367C mutation. Patterns of cartilage thickness differed between the genotypes at both ages studied. While average object thickness of the chondrocranium did not differ between in *Fgfr3* ^*Y367C/+*^ embryos relative to *Fgfr3* ^*+/+*^ littermates at E14.5, thickness mapping (Figure 7a–d) showed localized regions of difference, specifically in the nasal capsule, lower lateral walls, otic capsules, and most posterior aspect of the cranial base. By E16.5, average object thickness of the chondrocranium was significantly higher in *Fgfr3* ^*Y367C/+*^ embryos relative to *Fgfr3* ^*+/+*^ littermates. Thickness mapping at E16.5 showed increased cartilage thickness in the posterior nasal capsule, otic capsules, and posterior cranial base in *Fgfr3* ^*Y367C/+*^ embryos relative to *Fgfr3* ^*+/+*^ littermates (Figure 7e–h).

Although there was no difference in MC thickness as determined by average object thickness at E14.5, a thickened MC of reduced length was revealed in *Fgfr3* ^*Y367C/+*^ embryos as compared to *Fgfr3* ^*+/+*^ littermates at E16.5. Additionally, thickness mapping indicated a trend toward increased thickness of the medial regions of the left and right rods (arms) of Meckel's cartilage. By E16.5 there is an obvious difference in MC thickness between the genotypes along the length of MC, with increased thickness in the region of MC referred to as Mid1 (Pitirri et al., 2022; Svandova et al., 2020), a region often among the first to degrade as development progresses and MC begins to disappear. The thickened MC of reduced length may be attributed to the gain‐of‐function mutation of *Fgf3*, which reduces the differentiation of prehypertrophic chondrocytes into hypertrophic chondrocytes (Zhou et al., 2015). The decrease in the length of MC could also be explained by a reduction of the region that includes hypertrophic chondrocytes. It has been demonstrated that the size of the hypertrophic zone in MC of *Fgfr3* ^*Y367C/+*^ mice is shortened, and individual hypertrophic chondrocyte size is reduced (Duplan et al., 2016). It is well known that a paucity of fully enlarged hypertrophic chondrocytes is considered the principal factor of longitudinal growth in endochondral bones (Zhou et al., 2015).

In typically developing individuals, FGFR3 signaling is known to affect proliferation and differentiation of chondrocytes, matrix synthesis, and bone growth in developing growth plates (Arikawa‐Hirasawa et al., 1999; Li et al., 1997; Naski et al., 1998). FGFR3 is transduced through the RAS‐RAF1‐MEK1/2‐ERK1/2 pathway (RAS‐MAPK pathway) in a ligand (FGF)‐dependent manner (Eswarakumar et al., 2005; Yadav et al., 2012). The cellular mechanism of disease in ACH is associated with the amino acid substitution in the transmembrane domain of FGFR3, which results in activation of the RAS‐MAPK pathway in both ligand‐dependent and ‐independent manners. As a consequence, an excessive inhibition of chondrocyte proliferation and differentiation leads to impaired matrix synthesis and bone growth (Ornitz & Marie, 2015, 2019). Previous investigation of mandibular shape, size, and position in pediatric ACH patients revealed the mandible as a whole, or subunits such as the mandibular body and ramus were reduced in size at all ages (Duplan et al., 2016).

Our findings are relevant to the fields of 3D imaging, craniofacial and lower jaw development, and disease, and confirm early dysmorphology of the chondrocranium, which precedes that of MC shortening. The tight temporal control allowed in murine experiments reduces the variation introduced into experiments and enhances our understanding of important developmental processes (Musy et al., 2018; Pitirri et al., 2020), while 3D imaging and analyses provide information pinpointing specific, localized morphological and growth differences between *Fgfr3* ^*Y367C/+*^ embryos as compared to *Fgfr3* ^*+/+*^ littermates. Further exploration of the embryonic development of cartilage and the impact of currently available and upcoming treatments on the growth of the skull, development of the foramen magnum, and effects on the mandible (dentary) is needed to advance treatment of ACH.

## AUTHOR CONTRIBUTIONS

**Susan M. Motch Perrine:** Conceptualization; data curation; formal analysis; investigation; methodology; project administration; software; supervision; validation; visualization; writing – original draft; writing – review and editing. **Nishchal Sapkota:** Methodology; resources; software; validation; writing – original draft; writing – review and editing. **Kazuhiko Kawasaki:** Methodology; writing – original draft; writing – review and editing. **Yejia Zhang:** Software; validation; writing – review and editing. **Danny Z. Chen:** Funding acquisition; methodology; resources; supervision; validation; writing – review and editing. **Mizuho Kawasaki:** Methodology. **Emily L. Durham:** Methodology; writing – original draft; writing – review and editing. **Yann Heuzé:** Formal analysis; funding acquisition; validation; writing – original draft; writing – review and editing. **Laurence Legeai‐Mallet:** Methodology; writing – original draft; writing – review and editing. **Joan T. Richtsmeier:** Conceptualization; funding acquisition; project administration; resources; writing – original draft; writing – review and editing.

## Data Availability

Data are available through Penn State University Data Commons at https://www.datacommons.psu.edu/commonswizard/MetadataDisplay.aspx?Dataset=6367 (doi:10.26208/3HY4‐6S34) and include: 3D isosurfaces of chondrocranium and MC segmented from PTA enhanced microCT images at E14.5 and E16.5 and 3D landmark coordinates. Information on how to download the WinEDMA programs can be found at https://getahead.la.psu.edu/resources/edma and the EDMAinR programs are available on GitHub at https://github.com/psolymos/EDMAinR. Code for automatic chondrocranium segmentation with very sparse annotation via uncertainty‐guided self‐training is available through https://github.com/ndcse-medical/CartSeg_UGST. PTA staining protocols for various embryonic ages of mice are available: https://doi.org/10.1002/dvdy.136. Mice were previously published (Pannier et al., 2009).
